# A case report on serendipitous diagnosis of wilson’s disease in a child with brucellosis and pseudomonal infection

**DOI:** 10.1002/ccr3.4178

**Published:** 2021-05-06

**Authors:** Kiran Malbul, Srijana Katwal, Saurav Khetan, Nirjala Aryal

**Affiliations:** ^1^ Nepalese Army Institute of Health Sciences College of Medicine Kathmandu Nepal; ^2^ Department of Pediatrics Shree Birendra Hospital Kathmandu Nepal

**Keywords:** brucellosis, coinfection, D‐penicillamine, pediatric, rifampin, wilson's disease

## Abstract

Wilson's disease can have varied clinical manifestations and initial presentation can be misleading as in our case. Our case depicts the necessity of suspicion of WD in variable presentation of liver disorders, especially in pediatrics population.

## Graphical abstract

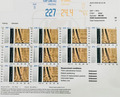

## 1 INTRODUCTION

Wilson's disease (WD) is a rare inherited autosomal recessive genetic defect of the copper metabolism. It frequently leads to progressive hepatolenticular degeneration and characterized mainly by hepatic and neurological manifestations.^1^ The prevalence is approximately one case per 30,000 live births^2^ with estimated high prevalence in the East Asian region.^3^ There is a mutation in ATP7B gene that encodes a copper transport protein on chromosome 13^4^, ^5^ leading to accumulation of copper into different tissues,^1^ resulting in various clinical manifestations predominantly hepatic, neurological, and psychiatric with at times the presence of Kayser‐Fleischer (KF) rings on the cornea, patients often present with a combination of symptoms.^6^, ^7^, ^8^ Early clinical features are nonspecific and sometimes maybe misleading in establishing a definitive diagnosis especially in younger patients and with concurrent other infections. Therefore, in young patients with unclear liver disease, hematologic or neurologic disorders, a differential diagnosis of Wilson's disease must be excluded.^6^ We report a case of Wilson's disease in a 9 year‐old girl presented initially with fever and progressive yellowish discoloration of the body, later confirmed to be having acute on chronic hepatic Wilson's disease with brucellosis and culture‐positive *Pseudomonas* infection and also illustrate the complexity of diagnosing Wilson's disease in the early stages of a clinical course complicated with infection.

## 2 CASE PRESENTATION

A 9 year‐old girl was admitted to this hospital for evaluation of fever and jaundice. Two days before this admission, the patient was taken to a local hospital where she was found to have severe anemia with a hemoglobin of 3.4 gm/dl. She was suspected and worked up on the line of hemolytic condition for which two units of packed RBCs were transfused, and she was referred to our center for further evaluation.

The patient had been in her usual state of health until 10 days before this admission, when she was noted to have intermittent fever with chills and rigor, maximum documented being 38.9^°^C, yellowish discoloration of skin, and dark‐colored urine. She also had right upper abdominal pain but no nausea, vomiting, hematemesis, hemoptysis, pruritus, passage of clay‐/dark‐colored stool, skin rashes, or joint pain.

The patient had been delivered vaginally at full term. Subsequent growth and development were reportedly normal, and her immunizations were up to date. She had no known medical conditions, was not taking any medications, and had no known allergies to medications. She lived in a family with no history of consanguinity, and they were involved in cattle rearing.

On examination, she was ill appearing and appeared tired. The temperature was 38.3^°^C, the heart rate 92 per minute, the blood pressure 90/60 mmHg, the respiratory rate 22 per minute, and the oxygen saturation 98% while breathing ambient air. The weight was 26 kg (25.78th percentile), and height was 134 cm (55.5th percentile). She had conjunctival icterus and pallor but no clubbing, palmar erythema, cyanosis, lymphadenopathy, and edema. The lungs were clear on auscultation. The abdomen was soft, with mild right upper quadrant tenderness, and liver was palpable 6 cm below the coastal margin with a span of 14 cm. Abdominal guarding, rebound tenderness, and Murphy's sign were absent. The spleen tip was palpated one cm below the left coastal margin. There was no fluid thrill or shifting dullness. The skin examination revealed no lesions, rash, abnormal pigmentation, or abnormality of the nails. She was alert and oriented and followed commands. The remainder of the examination was normal.

## 3 INVESTIGATIONS

**FIGURE 1:**
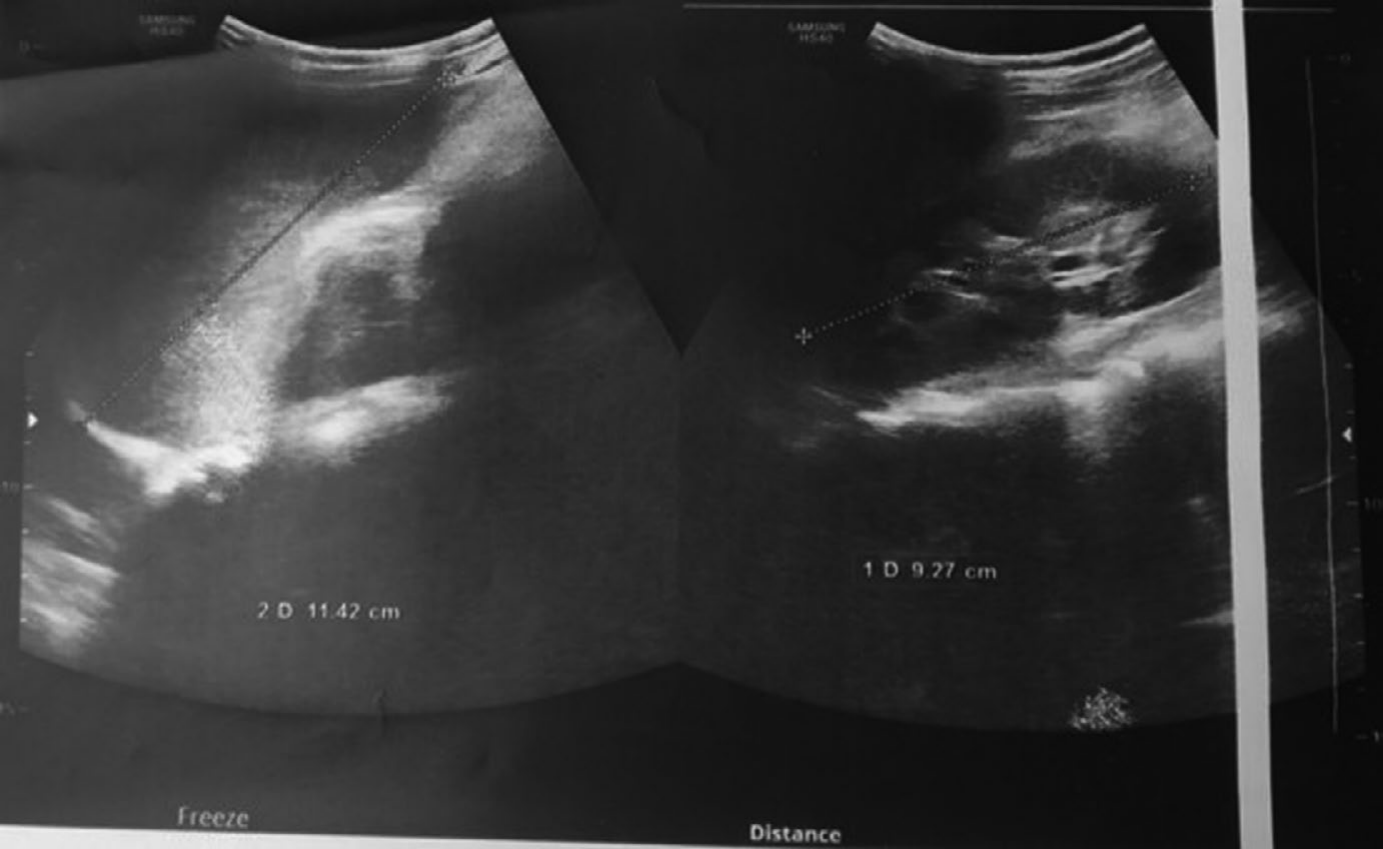
Ultrasonography showing hepatomegaly with coarse echotexture as compared to renal cortex

Routine laboratory testing obtained 2 days after original blood transfusion revealed a hemoglobin of 8.2 gm/dl, total bilirubin of 16.3 mg/dl, prothrombin time of 18.3 seconds, and activated partial thromboplastin time (aPTT) of 65 seconds. Blood levels of aspartate aminotransferase (AST), alanine aminotransferase (ALT), total protein, and albumin were normal. A direct Coombs test was positive, and tests for hepatitis A, B, and C viruses were negative. Remainder of the laboratory test results are shown in (Table 1). Ultrasound of abdomen revealed an enlarged liver with coarse echotexture, mild splenomegaly, and minimal ascites (Figure 1). Intravenous fluids and empiric antibiotics were started for fever, jaundice, and abdominal pain of unknown causation.

**TABLE 1:** Clinical laboratory results

Laboratory investigations	Illness Day 10, Hospital Day 1	Illness Day 12, Hospital Day 3	Illness Day 14, Hospital Day 5	Illness Day 19, Hospital Day 10	Illness Day 23, Hospital Day 14 (rifampin withdrawn)	Illness Day 26, Hospital Day 17	Illness Day 32, Hospital Day 23 (D‐penicillamine started)	Illness Day 49, Hospital Day 40	Reference range
White blood cell count (cells/**μl**)	7000	7400	8100	8600	9500	6700	4300	6020	4500‐11000
Hemoglobin (g/dl)	8.2↓	7.5↓	8.4↓	9.5↓	10↓	9.9↓	9.6↓	11	11.5‐15.5
Bilirubin, total (mg/dl)	16.3↑	2.1↑	2.17↑	2.77↑	5.13↑	2.91↑	1.92↑	1.3↑	0.2‐1.0
Bilirubin, conjugated (mg/dl)	6.9↑	1.1↑	1.4↑	1.88↑	3.27↑	1.82↑	1.13↑	0.5↑	<0.35
Aspartate aminotransferase (U/L)	28	76↑	75↑	86.6↑	336↑	263↑	113↑	60↑	15‐40
Alanine aminotransferase (U/L)	25	52↑	64↑	68.7↑	237↑	205↑	89↑	40↑	10‐36
Alkaline phosphatase (U/L)	29↓	53	57	117.2	158↑	130↑	137↑	114	30‐120
Prothrombin time (sec)	18.3↑	20↑	24.6↑	26.4↑	28.3↑	36.1↑	40↑	22↑	12.2‐15.5
International normalized ratio	1.2	1.4↑	1.6↑	1.8↑	1.9↑	2.3↑	2.6↑	1.5↑	0.8‐1.2
Platelet count (/mm3)	249 × 10^3^	191 × 10^3^	200 × 10^3^	240 × 10^3^	320 × 10^3^	300 × 10^3^	343 × 10^3^	182 × 10^3^	150‐450 × 10^3^
Activated partial thromboplastin time (sec)	65↑	63↑	68↑	70↑	73.6↑	53.5↑	86↑	63.3↑	26.5‐35.5

Note

↑ The value in the patient was above normal reference range.↓ The value in the patient was below normal reference range.

On day three of admission, *Brucella* serology was reported to be high at 1:160 (significant titer >=1:80) and blood culture grew *Pseudomonas aeruginosa*. At that moment, her AST was 76 U/L, ALT 52 U/L, and total bilirubin 2.1 mg/dl. The PT and aPTT were also elevated at 20 seconds and 63 seconds, respectively. Antibiotics were switched to cefepime, doxycycline, and rifampin. On day five of admission, she was transfused with another two units of packed RBC for a hemoglobin of 8.4 gm/dl. Total bilirubin decreased briefly after starting antibiotics but later on liver function continued to worsen with rising total bilirubin, indirect bilirubin, transaminases, and PT/INR. Because of worsening liver function, rifampin was discontinued on day fourteen of admission.

**FIGURE 2:**
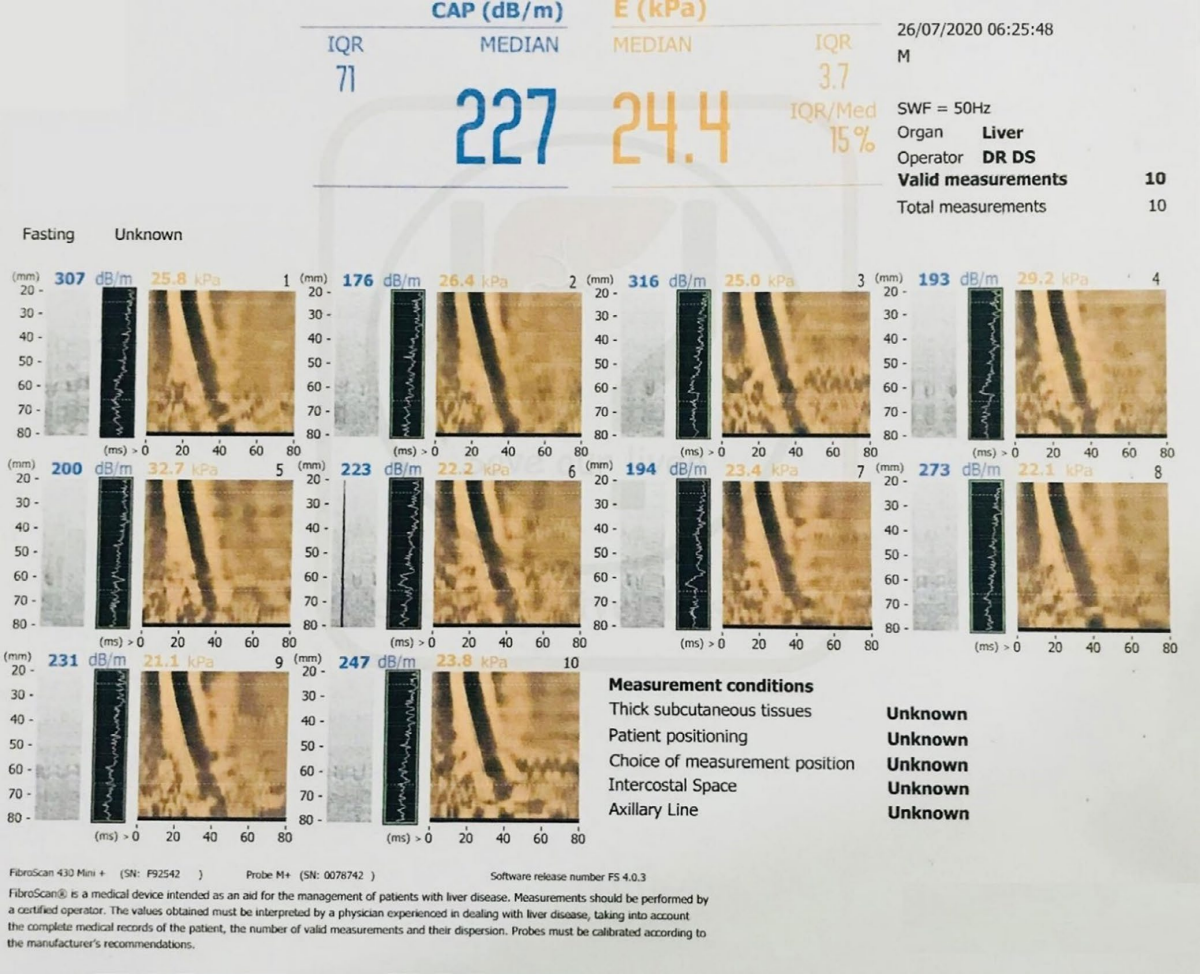
Fibroscan of liver showing advanced liver scarring

On day twenty second of admission, serum ceruloplasmin was found to be low at 9.18 mg/dl (reference range, 23‐51 mg/dl). The urinary copper excretion was elevated at 2069 microgram/day (reference range, 3‐50 microgram/day). Eye examination did not reveal KF ring. Results of ANA (Anti‐Nuclear Antibody) and anti‐dsDNA (double‐stranded deoxyribonucleic acid) were unremarkable. A diagnosis of Wilson's disease was made based on low ceruloplasmin and high urinary copper excretion, and she was started on D‐penicillamine at 20 mg/kg/day along with zinc and pyridoxine. Subsequent Fibroscan showed a fibrosis score of 24.4 kPa suggestive of advanced liver scarring (Figure 2). Following treatment with D‐penicillamine, liver function continued to improve, and she was discharged on day 40.

## 4 OUTCOME AND FOLLOW‐UP

Patient's clinical conditions improved with normalization of laboratory parameters mainly liver function tests. Subsequently, she was discharged with medical therapy with a plan to follow up after 3 months. They were counseled on nature of this disease, dietary restrictions, importance of extra vaccination, and side effects of the medications. Parents were advised for laboratory and genetic screening; however, they refused because of economical constraint, though they were screened for KF rings which were absent.

## 5 DISCUSSION

Wilson's disease is a rare autosomal recessive disorder usually presenting as early as 3 years to as late as 55 years of age^9^ and has varied clinical manifestations ranging from asymptomatic to hepatic, neurological, psychiatric, hematological, and ophthalmological symptoms.^1^, ^8^, ^9^ Given this wide variability in presentation, diagnosis of Wilson's disease can be challenging.

Children with Wilson's disease are usually normal at birth and may remain for a variable period of time.^10^, ^11^ Given defective protein resulting from ATPB7 gene mutation, copper transport across hepatocytes and subsequent biliary excretion becomes impaired.^4^ This leads to progressive copper accumulation within the hepatocytes, eventually exceeding hepatic storage capacity resulting in spillage of copper out of liver into the circulation which then gets gradually deposited in extrahepatic tissues like brain and Descemet's membrane of cornea.^12^, ^13^ Thus, liver manifestations are more common in first decade of life whereas neurological manifestations are common in third decades.^1^, ^14^ Liver is presumed to have considerable capacity to store excess copper, hence clinical manifestations are barely observed before 3 years of age.^9^, ^15^ Data from numerous pediatric series have shown 9–10 years as the average age of diagnosis of the disease.^7^

Not all cases of Wilson's disease have KF ring or present with typical hepatic, neurological, and psychiatric manifestations. Various studies have suggested that clinical and laboratory parameters only are not sufficient to make a diagnosis of Wilson's disease in patients with liver disease of unknown origin.^9^, ^13^, ^16^ In a series of cases studied by Nina Manolakit et al (2009), out of 57 cases, 25% presented with jaundice, 12% with acute Coombs negative hemolytic anemia, and only 38% of patient had KF ring.^17^ Similarly in a study done by Steindl et al (1997), out of 55 cases, serum ceruloplasmin level was <20 mg/dL in 73%, urinary copper excretion was increased in 88%, and liver copper content was elevated in 91% at diagnosis. KF rings were detected only in 55% of cases. In contrast to patients with neurological disease (90% KF rings, 85% low ceruloplasmin), only 65% of patients presenting with liver disease were diagnosed by these typical findings.^16^

In some cases, Wilson's disease becomes an incidental finding being discovered during work‐up of other health issues like infections.^7^ Our patient presented with acute onset of fever, jaundice, right upper quadrant abdominal pain and was found to have hepatosplenomegaly on physical exam. Initial laboratory work was positive for DCT negative hemolytic anemia, *Pseudomonas* bacteremia, and positive *Brucella* serology. Exposure to milked animals including a bovine calf raised the suspicion of Brucellosis whereas source of *Pseudomonas* bacteremia was not entirely clear. Nevertheless, she was treated with antibiotics targeting both *Brucella* and *Pseudomonas*, following which she defervesced, icterus diminished but she experienced few episodes of nausea, vomiting, and loss of appetite in between. Liver function continued to get worse despite appropriate antibiotic treatment which raised the possibility of other causes of liver failure including autoimmune liver disease and Wilson's disease. Based on a low serum ceruloplasmin and high urinary copper excretion, diagnosis of Wilson's disease was established and improvement of liver function test following treatment with D‐penicillamine and zinc gave further credence to the diagnosis of Wilson's disease. Similar therapeutic response has been reported in the study done by Chaudhari S et al wherein 45 out of 50 cases showed satisfactory response to D‐ penicillamine and zinc therapy.^14^

In children, hemolysis may be an initial presentation of Wilson's disease which might apparently get precipitated by infections or drugs and mostly is transient and self‐limiting, resulting in brief episodes of jaundice.^7^ Our patient might have been in latent phase of Wilson's disease till she got infected with brucellosis. The infection might have triggered DCT negative hemolytic anemia resulting in progressive jaundice, and antibiotics treatment might have initially lowered the severity of hemolysis leading to apparent decrease in initial bilirubin but later worsening of liver function test could have been from use of hepatotoxic drugs like doxycycline^18^, ^19^ and rifampin.^20^, ^21^ She had significant fibrosis of liver on Fibroscan suggestive of chronic liver damage and was likely more susceptible to hepatotoxic effect of medications. She had no neurological symptoms, and KF rings were also absent. This is in accordance with the study done by Kalamar et al where a patient presented with hepatic lesions but did not have KF ring and neurological manifestations.^22^

A positive family history, detectable KF rings, low ceruloplasmin level (<20mcg/dL), elevated free copper >25 mcg/dL, and 24 hours urine copper >100 mcg/24 hours are some parameters which can help to make a diagnosis of Wilson's disease.^23^ Hepatic copper concentration and ATP7B gene sequencing are the gold standard tests for definitive diagnosis of Wilson's disease.^24^ However, diagnosis is mainly based on combination of clinical features, laboratory results, and mutation analysis.^13^, ^17^ Scoring systems like modified Leipzig score can be useful in patients presenting with liver disease with inconclusive diagnosis where a cumulative score of four or above establishes the diagnosis of Wilson's disease.^8^ Findings suggestive of chronic liver inflammation on ultrasound abdomen and Fibroscan along with a score of five on modified Leipzig score strongly suggested the diagnosis of Wilson's disease.^25^, ^26^, ^27^ Ultrasound and Fibroscan findings, however, need to be interpreted with caution. Ultrasound of liver can be useful in differentiating acute hepatitis from chronic one as an irregular liver capsule, and coarse echotexture may suggest chronic liver disease, as opposed to acute severe hepatitis. Noninvasive tests like Fibroscan assessing the degree of liver scarring can be a reliable method of detecting liver fibrosis and thereby helping to differentiate acute and chronic hepatitis but accuracy of such scan in pediatric population with age less than 12 years remains uncertain.

## 6 CONCLUSION

In summary, Wilson's disease is a treatable disease which needs clinical focus with combined investigations to diagnose it on time and prevent mortality and morbidity from it, especially in pediatrics population with variable presentation of liver disorders. It also encourages us to be more cautious in using hepatotoxic medications in cases of diagnosed or suspected liver disorders like Wilson's disease.

## CONFLICT OF INTEREST

None declared.

## AUTHOR CONTRIBUTIONS

**Kiran Malbul and Srijana Katwal** were involved in conception of the study, acquisition of data, drafting and reshaping the initial manuscript, and revising the contents.

**Saurav Khetan** was involved in acquisition of data and reshaping the manuscript whereas **Nirjala Aryal** helped in revising the manuscript critically for important intellectual content.

All authors approved the final version of the manuscript and agreed to be accountable for all aspects of the work in ensuring that questions related to the accuracy or integrity of any part of the work are appropriately investigated and resolved.

## ETHICAL APPROVAL

Need for ethical approval waived. Consent from the patient's parents deemed to be enough.

## INFORMED CONSENT

Written informed consent was taken from the patient's parents before writing the manuscript.

## Data Availability

The data that support the findings of this study are available from the corresponding author upon reasonable request.
